# Timing of illumination is essential for effective and safe photodynamic therapy: a study in the normal rat oesophagus

**DOI:** 10.1038/sj.bjc.6690132

**Published:** 1999-02

**Authors:** J van den Boogert, R van Hillegersberg, H J van Staveren, R W F de Bruin, H van Dekken, P D Siersema, H W Tilanus

**Affiliations:** Laboratory for Experimental Surgery, Erasmus University, PO Box 1738, Rotterdam, 3000 DR, The Netherlands; Department of Surgery, University Hospital Rotterdam ‘Dijkzigt’, Dr. Molewaterplein 40, Rotterdam, 3015 GD, The Netherlands; Department of Clinical Physics, Daniel den Hoed Cancer Centre, Groene Hilledijk 301, Rotterdam, 3075 EA, The Netherlands; Department of Clinical Pathology, University Hospital Rotterdam ‘Dijkzigt’, Dr. Molewaterplein 40, Rotterdam, 3015 GD, The Netherlands; Department of Internal Medicine II, University Hospital Rotterdam ‘Dijkzigt’, Dr. Molewaterplein 40, Rotterdam, 3015 GD, The Netherlands

**Keywords:** 5-aminolaevulinic acid, oesophagus, illumination, nerve, photodynamic therapy, rat

## Abstract

5-Aminolaevulinic acid (ALA)-induced, protoporphyrin IX (PpIX)-mediated photodynamic therapy (PDT) is an experimental treatment modality for (pre)malignant oesophageal lesions. This study aimed to optimize the time of illumination after ALA administration. Six groups of eight rats received 200 mg kg^−1^ALA orally, eight rats served as controls. Illumination was performed at 1, 2, 3, 4, 6 or 12 h after ALA administration with a 1-cm cylindrical diffuser placed in a balloon catheter (laser parameters: 633 nm, 25 J radiant energy, power output 100 mW). During illumination, fluorescence measurements and light dosimetry were performed. Animals were sacrificed at 48 h (*n*= 4) or 28 days (*n*= 4) after PDT. At day 28, an oesophagogram was performed. Largest PpIX fluorescence was found at 3 h after ALA administration. In vivo fluence rate was three times higher than the calculated incident fluence rate. At 48 h after PDT, major epithelial damage was found in all animals illuminated at 2 h, whereas less epithelial damage was found at 3–6 h and none at 1 and 12 h. In animals illuminated at 4, 6 and 12 h, but not at 2 h, oesophagograms showed severe dilatations and histology showed loss of Schwann cells. These results demonstrate that the choice of time interval between ALA administration and illumination is critical for achieving epithelial damage without oesophageal functional impairment. A short interval of 2–3 h seems to be most appropriate. © 1999 Cancer Research Campaign


*British Journal of Cancer* (1999) **79**(5/6), 825–830
© 1999 Cancer Research Campaign
Article no. bjoc.1998.0132

### Timing of illumination is essential for effective and safe photodynamic therapy: a study in the normal rat oesophagus

**J van den Boogert[1], R van Hillegersberg[2], HJ van Staveren[3], RWF de Bruin[1], H van Dekken[4], PD Siersema[5] and HW Tilanus[2]**

[1]Laboratory for Experimental Surgery, Erasmus University, PO Box 1738, 3000 DR Rotterdam, The Netherlands; [2]Department of Surgery, University Hospital Rotterdam 'Dijkzigt', Dr. Molewaterplein 40, 3015 GD Rotterdam, The Netherlands; [3]Department of Clinical Physics, Daniel den Hoed Cancer Centre, Groene Hilledijk 301, 3075 EA Rotterdam, The Netherlands; Departments of [4]Clinical Pathology and [5]Internal Medicine II, University Hospital Rotterdam 'Dijkzigt', Dr. Molewaterplein 40, 3015 GD Rotterdam, The Netherlands

**Summary** 5-Aminolaevulinic acid (ALA)-induced, protoporphyrin IX (PpIX)-mediated photodynamic therapy (PDT) is an experimental treatment modality for (pre)malignant oesophageal lesions. This study aimed to optimize the time of illumination after ALA administration. Six groups of eight rats received 200 mg kg$^{-1}$ ALA orally, eight rats served as controls. Illumination was performed at 1, 2, 3, 4, 6 or 12 h after ALA administration with a 1-cm cylindrical diffuser placed in a balloon catheter (laser parameters: 633 nm, 25 J radiant energy, power output 100 mW). During illumination, fluorescence measurements and light dosimetry were performed. Animals were sacrificed at 48 h ($n$ = 4) or 28 days ($n$ = 4) after PDT. At day 28, an oesophagogram was performed. Largest PpIX fluorescence was found at 3 h after ALA administration. In vivo fluence rate was three times higher than the calculated incident fluence rate. At 48 h after PDT, major epithelial damage was found in all animals illuminated at 2 h, whereas less epithelial damage was found at 3–6 h and none at 1 and 12 h. In animals illuminated at 4, 6 and 12 h, but not at 2 h, oesophagograms showed severe dilatations and histology showed loss of Schwann cells. These results demonstrate that the choice of time interval between ALA administration and illumination is critical for achieving epithelial damage without oesophageal functional impairment. A short interval of 2–3 h seems to be most appropriate.

**Keywords:** 5-aminolaevulinic acid; oesophagus; illumination; nerve; photodynamic therapy; rat

The incidence of oesophageal adenocarcinoma has been rising more rapidly over the past decade than that of any other solid tumour (Blot et al, 1991). The majority of these carcinomas arises in a Barrett's oesophagus, which is characterized by metaplastic specialized columnar epithelium (Cameron et al, 1995). The development of cancer is thought to progress morphologically through low- and high-grade dysplasia to invasive carcinoma. Oesophagus resection has been advocated for patients with high-grade dysplasia. However, this is controversial because its associated morbidity and mortality have been judged too high in context of a preneoplastic disease (Wang, 1994). A less mutilating treatment is required that would be acceptable to more patients and applicable in patients at high risk for surgery and general anaesthesia. Photodynamic therapy (PDT) could be such a treatment as it ideally causes only necrosis of the mucosa and submucosa, leaving the muscularis propria intact. Large areas of tissue can be treated and general anaesthesia is not required. PDT is a technique combining photosensitizing agents and illumination in the presence of tissue oxygen to produce photochemical tissue destruction. In combination with acid suppression, PDT may lead to re-epithelialization with normal-appearing squamous epithelium. In some cases, however, islands of residual columnar epithelium and thus of potentially premalignant cells have been described underneath regenerated squamous mucosa, so-called pseudoregression (Laukka and Wang, 1995; Barr et al, 1996). These therapy-resistant cells may be eliminated by improving the PDT treatment.

In this study, the optimal time of illumination after administration of 5-aminolaevulinic acid (ALA) was determined in a rat model. The effectiveness of destroying epithelial cells as well as the possible impact on oesophageal function in the healing phase were evaluated.

*Received 27 March 1998*
*Revised 19 June 1998*
*Accepted 24 July 1998*

*Correspondence to:* J van den Boogert, Laboratory for Experimental Surgery, Erasmus University, PO Box 1738, 3000 DR Rotterdam, The Netherlands

### MATERIALS AND METHODS

### Animals

The experimental protocol was approved by The Committee in Animal Research of the Erasmus University. Sixty-two male Wistar rats (Harlan CPB, Zeist, The Netherlands) weighing 275–325 g were used. They had free access to tap water and rat chow (AM II, Hope Farms, Woerden, The Netherlands). To avoid skin lesions, the animals were put in a cage in subdued light.

### Experimental design

Fifty-six animals were randomly allocated to seven groups of eight animals each. In six treatment groups, the animals received 200 mg kg$^{-1}$ ALA (Sigma Chemical, St Louis, MO, USA) dissolved in phosphate-buffered saline, by single oral gavage. The

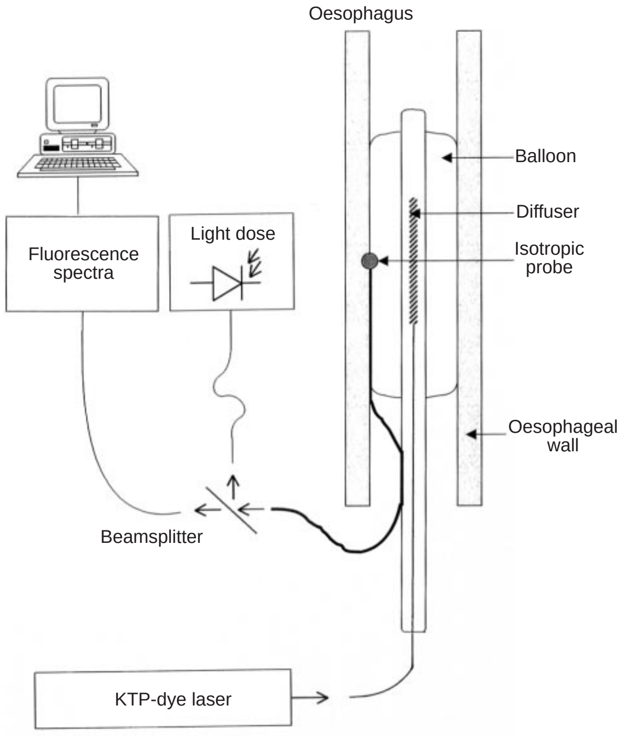

**Figure 1** Experimental set-up for oesophagus PDT facilitating a homogeneous illumination and real-time measurements of the true light fluence rate and PpIX fluorescence spectra. Illumination parameters: 1 cm cylindrical diffuser, 25 J radiant energy, power output 100 mW

animals in the seventh group served as controls and received phosphate-buffered saline only. PDT was carried out under intramuscular ketamine and xylazine anaesthesia. In the six treatment groups, illumination was performed at 1, 2, 3, 4, 6 or 12 h after ALA administration. In control animals, illumination was performed at 3 h. Four rats in each group were sacrificed at 48 h after illumination to study the acute effects, as a pilot study had shown a maximum PDT effect at 48 h. The other four rats in each group were sacrificed on day 28 after illumination to study healing because, by then, a pilot study had shown complete re-epithelialization. Furthermore, that time interval was found to show full expression of stenosis after oesophageal corrosive burns (Berthet et al, 1994).

Additionally, six animals were used for separate temperature and pressure measurements and were sacrificed 48 h after the procedure.

### Light delivery

A balloon catheter (PTA Balloon Catheter Opta 5, Cordis, Roden, The Netherlands) was used to deliver homogeneous and circumferential light to the oesophagus (Panjehpour et al, 1993). The device consisted of a semiflexible catheter and an inflatable cylindrical optically clear balloon (length 20 mm; outside diameter 3.5 mm inflated with 0.4 ml air) attached to the distal end of the catheter (Figure 1). A 400-μm fibre with a 10-mm length cylindrical diffusing tip of 760 μm diameter (Lightstic 360, Rare Earth Medical, West Yarmouth, MA, USA) was placed exactly in the centre of the balloon and thus centrally in the oesophagus.

Light of 633 nm (600 Series Dye Module pumped by KTP/532 laser, Laserscope, San Jose, CA, USA) was transmitted through the fibre. A spectroscope (WaveMate, Coherent, Auburn, CA, USA) was used to verify the accuracy of the laser wavelength. The output power emitted by the fibre tip, 100 mW, was calibrated and measured before and after treatment by the built-in power meter of the dye laser.

Illumination was performed with a radiant energy of 25 J, giving an incident light dose of approximately 22.7 J $cm^{-2}$ tissue surface (area of a 1-cm long, 3.5-mm diameter cylinder).

### Fluorescence and dosimetry

True light fluence (J $cm^{-2}$) and protoporphyrin IX (PpIX) fluorescence spectra were monitored during illumination at 15 s intervals with a spherical isotropic probe of 800 μm diameter (Rare Earth Medical), which was positioned halfway along the cylindrical diffuser and pressed against the oesophageal wall by the balloon catheter (Figure 1). The distal fibre end was led to a 50%/50% beam splitter (Rare Earth Medical) to transport part of the light to a light-dose meter and the other part to an optical multichannel analyser system (Multispec 77400 spectrometer + Instaspec-IV 77131 CCD-camera, Oriel Instruments, CT, USA) with a built-in combined 630-nm notch plus 665-nm long wavelength pass filter to block the laser light. The response of the isotropic probe was calibrated for true fluence rate measurements using an integrating sphere with a homogeneous diffuse light field (Van Staveren et al, 1995).

Recorded in vivo fluorescence spectra were normalized by dividing them by the fluence rate, which was simultaneously recorded by the light-dose meter. This yields, in first order approximation, spectra that are corrected for different tissue optical properties, geometrical aberrations and different output power of the cylindrical diffuser. Next, the mean normal tissue autofluorescence spectrum measured in the control group was subtracted from the fluorescence spectra measured in the animals with oral ALA administration. The remaining PpIX fluorescence peak was integrated over a 15-nm range resulting in a number that is a measure for the average local PpIX concentration.

### Measurement of temperature and pressure effect

To monitor the thermal effects of the procedure, temperature was measured every 10 s in three rats during illumination at 3 h after administration of ALA. Three thermal probes (SMM probe for model 790 fluoroptic thermometer, Luxtron, Santa Clara, CA, USA) were placed either in the suprahepatic space or supragastric space next to the oesophagus, or in the oesophagus between the inflated balloon and the oesophageal wall.

The effect of pressure from the inflated balloon was studied by histological examination of the oesophagus of three rats, 48 h after a sham procedure in which the balloon was inflated during 250 s without illumination and without previous ALA administration.

### Analysis of tissue damage

In rats sacrificed 48 h after PDT, the oesophagus with a small piece of stomach was removed. The oesophagus was opened longitudinally, the circumference at the laser site was measured and the macroscopic and microscopic appearance were noted. Thereafter, the oesophagus was curled up from distal to proximal, fixed in formalin, sectioned and stained with haematoxylin and eosin for conventional light microscopy. Damage was scored

 *© Cancer Research Campaign 1999*

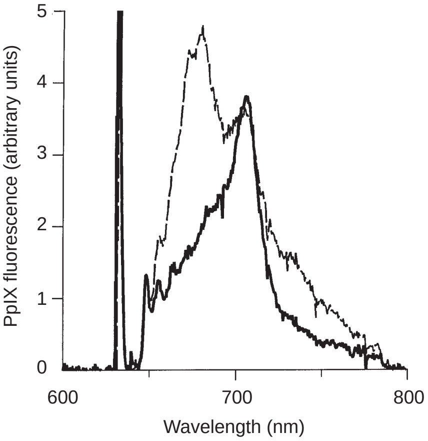

**Figure 2** Typical PpIX fluorescence (—) with a peak at 705 nm and anomalous fluorescence with a peak at 680 nm observed in five animals (- - -). Spectra are corrected for the normal tissue autofluorescence

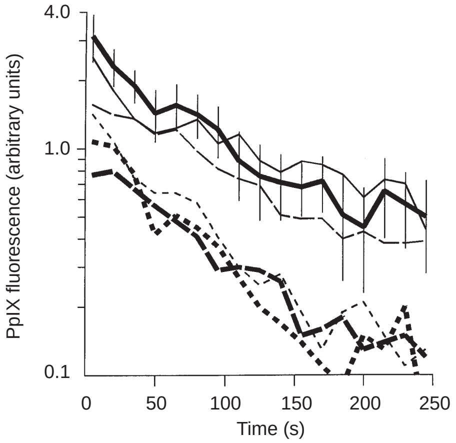

**Figure 3** Mean integrated PpIX fluorescence at 705 nm during optical irradiation. The error bars are standard errors of the mean and are indicative of the interanimal variation ($n$ = 8 for each group). Time of illumination after ALA administration: 1 h (▬ ▬); 2 h (— —), 3 h (▬▬), 4 h (——), 6 h (▪ ▪ ▪) and 12 h (- - -)

quantitatively on a scale from 0 to 3 for each separate oesophageal layer by a pathologist who was not aware of the performed treatment. Oedema of the submucosa and serosa was scored based on the thickness of the submucosa (–, normal; +, 2 times thicker than normal; ++, 3–5 times thicker; +++, ≥ 5 times thicker). Inflammation of the submucosa, muscularis propria and serosa was scored on the basis of the amount of inflammatory cells (mostly lymphocytes) using a grid (–, none; +, < 1 per grid; ++, 1–2 per grid; +++, ≥ 3 per grid). The severity of the necrosis of the muscularis propria was scored on basis of the presence of vital muscle cells (–, 100%; +, > 75%, ++, > 25%, +++, ≤ 25%). In rats sacrificed at day 28 after PDT, a barium oesophagogram under intramuscular ketamine (60 mg $kg^{-1}$) and xylazine (2.5 mg $kg^{-1}$) anaesthesia was performed before sacrifice to diagnose stenotic changes and dilatations. Thereafter, rats were sacrificed and the oesophagus was taken out, opened longitudinally and the circumference was measured at 0.5-cm intervals. Apart from the haematoxylin and eosin staining, the sections were stained with anti-S-100 (labels Schwann cells of the peripheral nervous system) to investigate possible changes in nerve tissue (Nada and Kawana, 1988). The number of anti-S-100 positive areas along the oesophagus was scored using a grid. Thereafter, the average number of anti-S-100 positive areas at the laser site and at the non-laser site was compared with oesophageal diameter on the oesophagogram.

### Statistical analysis

The values are expressed as mean ± standard error of the mean (s.e.m.). Comparisons of oesophageal circumference, diameter on oesophagogram, weight and fluorescence intensity were made using Student's $t$-test. Comparison of weight was controlled by the Mann–Whitney $U$-test. Spearman's rank order correlation for anti-S-100 staining against oesophageal diameter was used to analyse the relation between nerve damage and oesophageal diameter. A difference was considered to be significant at $P$-values of < 0.05.

### RESULTS

One animal (illuminated at 3 h after ALA administration) died of anaesthesia during illumination. One animal (illuminated at 6 h after ALA administration) died at day 7 after PDT of oesophageal necrosis and perforation.

### Temperature and pressure effects

Temperature of the suprahepatic and supragastric space maximally rose 0.6°C to a maximum of 36.7°C. The temperature in the oesophagus maximally rose 1.2°C to a maximum of 37.1°C. Histological analysis of the oesophagi of the rats after sham balloon insufflation procedure showed no abnormalities.

**Table 1** Histopathological changes of the oesophageal wall at 48 h after ALA-PDT in different treatment groups ($n$ = 4 for all groups)

| Illumination time after ALA (h) | Loss of epithelium ($n$) | Submucosa | | Muscularis propria | | Serosa | |
|---|---|---|---|---|---|---|---|
| | | Oedema | Inflammation | Inflammation | Necrosis | Oedema | Inflammation |
| Control | 0 | – | – | – | – | – | – |
| 1 | 0 | + | + | + | – | – | – |
| 2 | 4 | +++ | ++ | +++ | +++ | +++ | + |
| 3 | 1 | +++ | + | +++ | +++ | ++ | ++ |
| 4 | 1 | ++ | ++ | ++ | + | + | +++ |
| 6 | 1 | + | ++ | + | – | ++ | ++ |
| 12 | 0 | – | – | – | – | ++ | ++ |

Treatment parameters: 200 mg $kg^{-1}$ ALA, 1 cm cylindrical diffuser, 25 J radiant energy, 100 mW power output. Changes within layers: –, none; +, mild; ++, moderate; +++, severe (see Materials and methods section).

© Cancer Research Campaign 1999 

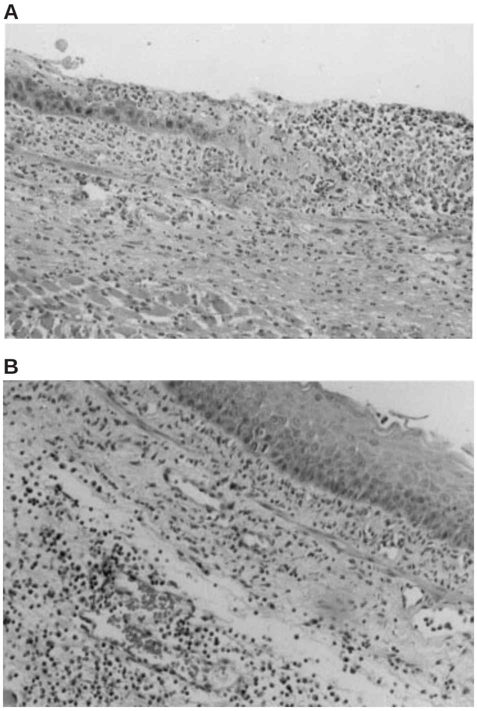

**Figure 4** Histopathological section of the rat oesophagus at the laser site 48 h after PDT with illumination at 2 h (**A**) and at 3 h (**B**) after administration of ALA. E, epithelium; LP, lamina propria, MM, muscularis mucosae; SM, submucosa; M, muscularis propria (× 20, haematoxylin and eosin stain)

**Table 2** Circumference and diameter of the oesophagus at the laser site and weight gain of the rats at 28 days after PDT (*n* = 3 for groups illuminated at 3 and 6 h, *n* = 4 for the other groups)

| Illumination time after ALA (h) | Circumference (mm ± s.e.m.) | Diameter on radiograph (mm ± s.e.m.) | Weight gain (g ± s.e.m.) |
|---|---|---|---|
| Control | 4.3 ± 0.3 | 3.3 ± 0.2 | 80 ± 5 |
| 1 | 4.5 ± 0.3 | 3.5 ± 0.2 | 74 ± 5 |
| 2 | 5 ± 0.3 | 4.3 ± 0.2* | 50 ± 4* |
| 3 | 5.7 ± 0.3* | 5.1 ± 0.2* | 65 ± 2* |
| 4 | 10.3 ± 0.8* | 6.4 ± 0.3* | – 2 ± 51 |
| 6 | 10 ± 1.7* | 6.0 ± 0.8* | – 32 ± 38* |
| 12 | 10 ± 1.1* | 6.3 ± 0.4* | 5 ± 39 |

**P*-value < 0.05 compared with the control group. Treatment parameters: 200 mg $kg^{-1}$ ALA, 1 cm cylindrical diffuser, 25 J radiant energy, 100 mW power output.

### Tissue fluorescence

The in vivo fluorescence spectrum recorded in rats after administration of ALA showed the PpIX-specific fluorescence peak at 705 nm, and was virtually the same as in the in vitro fluorescence of PpIX dissolved in Triton (2 nmol $ml^{-1}$) measured under the same conditions (Figure 2). The PpIX peak at 635 nm interferes with the illumination light (633 nm) and is blocked by the long wavelength pass filter. Besides the typical 705-nm PpIX fluorescence peak, an additional 680-nm peak was observed in five animals (one control, one at 1 h, one at 3 h, and two at 4 h after ALA administration), probably due to mucus, food components or bacteria (Figure 2). Largest initial PpIX fluorescence was observed in the group illuminated at 3 h after ALA administration (Figure 3). Rats illuminated at 1, 6 or 12 h after ALA administration showed a significantly smaller initial fluorescence peak ($P < 0.01$, $P < 0.02$, and $P < 0.02$ respectively) compared with values at 3 h, whereas animals illuminated at 2 and 4 h after ALA administration did not differ significantly ($P = 0.07$ and $P = 0.20$ respectively). Fluorescence intensity declined with illumination time to intensities of around background level after 150 s in the groups illuminated at 1, 6 and 12 h after ALA administration. In the other treatment groups, fluorescence was still declining at the end of illumination.

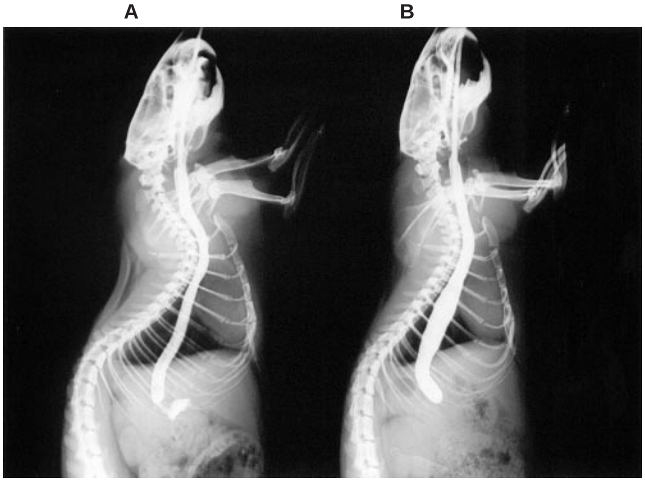

**Figure 5** Oesophagogram at 28 days after ALA-PDT: (**A**) illumination at 1 h after ALA, (**B**) illumination at 12 h after ALA. In rat (**A**), no dilatations nor strictures were seen. In rat (**B**), the oesophagus was dilated at the laser site with a doubling of the diameter. Arrows indicate the site of laser treatment

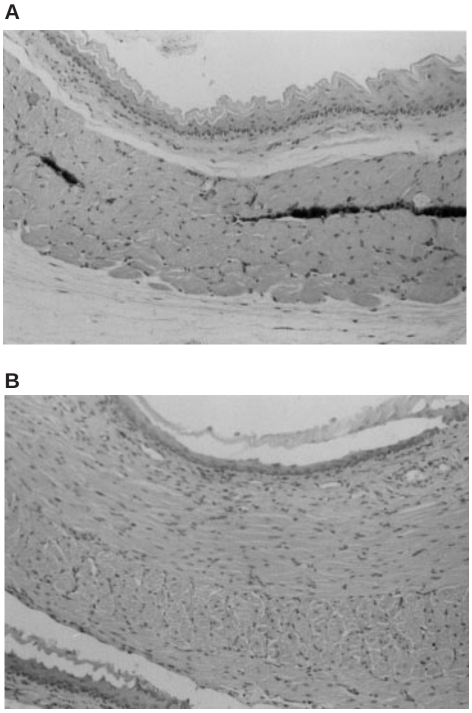

**Figure 6** Nerve tissue staining at the non-laser site (**A**) and at the laser site (**B**) 28 days after PDT with illumination at 12 h after administration of ALA. Arrows indicate the myenteric plexus, E, epithelium; SM, submucosa; M,

© Cancer Research Campaign 1999

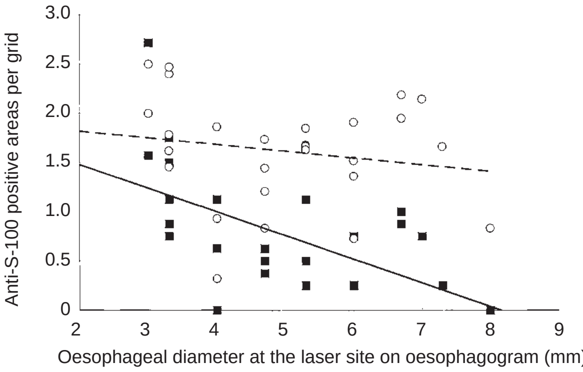

**Figure 7** Anti-S-100 staining at the laser site (■) and non-laser site (○) vs oesophageal diameter at the laser site on oesophagogram, together with the corresponding trendlines at the laser site (——) ($P$ = 0.02, $r$ = – 0.56), and at the non-laser site (- - -) ($P$ = 0.39, $r$ = – 0.17)

### Dosimetry

During optical irradiation, the true fluence rate was on average 3.0 ± 0.1 (mean ± s.e.m. $n$ = 56) times higher than the incident non-scattered irradiation, with a minimum of 1.4 and a maximum of 4.1.

### Histopathological changes 48 h after PDT

On microscopic examination, no abnormalities were seen in control animals (Table 1). Diffuse loss of epithelium in all animals was only seen in the group illuminated at 2 h after ALA administration. In this group, full thickness necrosis with acute inflammatory cell infiltration and diffuse swelling of the submucosa was observed. The epithelial damage was almost complete; however, some patches with one layer of epithelial cells were found, showing mitotic activity (Figure 4A). In the groups illuminated at 3, 4 or 6 h after ALA administration, loss of epithelium was only seen in one of four animals. In the other three, the epithelial layer was normal, with different degrees of submucosal oedema and inflammation and necrosis of the muscularis (Figure 4B). Illumination at 1 or 12 h after ALA administration did not cause any epithelial lesions.

### Histological and radiological changes 28 days after PDT

Twenty-eight days after illumination, 4 of 11 rats treated at 4, 6 or 12 h after ALA administration had lost more than 30% of their original weight (Table 2). However, no statistically significant difference in the groups illuminated at 4 or 12 h was reached because of large variations in gaining weight. In both groups, one animal lost and one animal gained considerable weight (4 h, loss 154 g and gain 59 g; 12 h, loss 102 g and gain 77 g). Weight in these four animals correlated negatively with the oesophageal circumference (4 h, –154 g/12 mm oesophageal circumference and +59 g/9 mm; 12 h, –102 g/11 mm and +77 g/7 mm). None of the animals showed any strictures on the oesophagogram. The oesophagus was significantly dilated at the laser site in all animals ($P$ < 0.02) except in the group treated at 1 h after ALA administration (Figure 5); almost a doubling of the circumference at the laser site was found in the groups treated at 4, 6 and 12 h ($P$ < 0.02) (Table 2). Microscopic examination showed complete re-epithelialization and a normal appearing submucosa, muscularis and serosa in all groups. Oesophageal diameter on the oesophagogram was significantly negatively correlated with the intensity of Schwann cell staining (anti-S-100) at the laser site ($P$ = 0.02, $r$ = –0.56) (Figures 6 and 7). No correlation was found between the intensity of Schwann cell staining and diameter at the non-laser site ($P$ = 0.39, $r$ = – 0.17).

### DISCUSSION

This study considers the efficacy and safety of ALA-PDT of the normal rat oesophagus. Maximal efficacy was found when rats were treated 2 h after oral administration of 200 mg kg$^{-1}$ ALA. Illumination at 4, 6 or 12 h after ALA administration caused oesophageal dilatation, functional impairment and weight loss.

In a previous study, we found that, after oral administration of 200 mg kg$^{-1}$ ALA, ALA-induced fluorescence of PpIX in the normal rat oesophagus was located almost exclusively in the basal cell layer of the oesophageal epithelium (Van den Boogert et al, 1997). Fluorescence of the submucosa and muscularis propria was near background levels. We, therefore, expected to find selective damage to the epithelial layer only. Diffuse destruction of the epithelium in all animals was found only in the group treated 2 h after ALA administration. Even in this group, small areas of epithelial cells showing mitotic activity remained after treatment. It is not clear whether the epithelium in these areas indicates re-epithelialization or incomplete epithelial damage, which may lead to pseudoregression as observed in clinical situations (Laukka and Wang, 1995; Barr et al, 1996). The present illumination schemes were chosen, based on results from pilot studies, with the aim of achieving a range of biological responses (mild to severe damage). Further alterations in the illumination schedule may possibly eliminate these remaining areas of viable epithelium. For example, a longer illumination time in the groups treated at 2–4 h could still increase the PDT effect because significant PpIX fluorescence was present at the end of illumination (Figure 3). Furthermore, adjustment of the power output, or fractionated illumination, may improve the efficacy of PDT (Messmann et al, 1995; Robinson et al, 1998).

In the groups illuminated at 1, 3, 4, 6 or 12 h after ALA, the oesophageal epithelium was mostly completely intact. However, in different degrees, damage to the submucosa, muscularis and serosa was always present (Table 1). One explanation for this damage pattern is that the photodynamic threshold (tissue photosensitizer concentration × true light dose) to induce necrosis is lower for muscle than for epithelium (Cowled and Forbes, 1985). One reason for that could be a difference in sensitivity to oxygen radicals (the damaging agents formed during PDT) between epithelium and muscularis. Several studies have suggested that muscle tissue is particularly prone to oxygen radical-mediated cell membrane damage (Jackson and O’Farrell, 1993). Non-protein sulphydryls in the epithelium are capable of binding free radicals, and rapid cell turn over and the process of restitution contribute to an intact epithelial lining (Forssell, 1988). Another explanation for the observed muscle damage could be a high production rate of haemoglobin and myoglobin in muscle. As part of this fast dynamic process, only few of the intermediates between ALA and haem will accumulate. This may lead to a low level of the intermediate PpIX and hence of a low fluorescence intensity on point fluorescence measurement. However, this may be translated into a high cumulative level of PpIX during PDT, resulting in a high production rate of singlet oxygen.

© Cancer Research Campaign 1999

Although we found extensive damage to the submucosa and muscle, at longer term we did not find any strictures. However, severe dilatations occurred in all rats treated 4, 6 or 12 h after ALA administration. This phenomenon could be explained by muscle fibre necrosis, a peripheral neuropathy or a combination of both. Muscle fibre necrosis seems unlikely because at 48 h after PDT muscle damage was most pronounced in the groups illuminated at 2 and 3 h after ALA administration. In this study, a significant relation between the absence of anti-S-100 staining and oesophageal dilatation at the treatment site was found, indicating nerve tissue damage in the myenteric plexus at the laser site. This may be caused by a relative late production of PpIX in nerve tissue, predominantly in Schwann cells rather than in nerve cells (Whetsell et al, 1978). Furthermore, the brain and nervous system are considered to be more susceptible to oxidative damage than other tissues because of their high content of polyunsaturated lipid-rich neural parenchyma, high oxygen consumption, and low content of scavengers (Urano et al, 1997). From an electron microscopic study of the normal mouse skin, it appeared that, besides endothelial cells and fibroblasts, nerve fibres are most sensitive to photodynamic therapy (using haematoporphyrin derivative) (Zhou et al, 1985).

This study confirms earlier studies by Murrer et al (1997) and Van Staveren et al (1994), i.e. that the true fluence rate in hollow organs is larger than the calculated incident fluence rate due to the strong light scattering nature of tissue. In a clinical setting, real-time light dosimetry and PpIX fluorescence measurements may be used to refine the PDT treatment by delivering equal light doses to different areas and monitoring PpIX levels to determine the duration of the illumination.

In conclusion, our results show that both the effectiveness and safety of ALA-PDT treatment for oesophageal lesions largely depend on the time between ALA administration and illumination. Further improvement of the illumination parameters leading to complete epithelial destruction is needed before it can be used as therapy for Barrett's oesophagus in clinical practice.

### ACKNOWLEDGEMENTS

This study was supported by a grant from The Netherlands Digestive Disease Foundation (WS 95-10). The authors thank Mr JHR Eikelaar for his assistance, Dr WCJ Hop for his advice concerning statistics and Dr RL Marquet for reading the manuscript.

### REFERENCES

Barr H, Shepherd NA, Dix A, Roberts DJH, Tan WC and Krasner N (1996) Eradication of high-grade dysplasia in columnar-lined (Barrett's) oesophagus by photodynamic therapy with endogenously generated protoporphyrin IX. *Lancet* **348**: 584–585

Berthet B, DiConstanzo J, Arnaud C, Choux R and Assadourian R (1994) Influence of epidermal growth factor and interferon γ on healing of oesophageal corrosive burns in the rat. *Br J Surg* **81**: 395–398

Blot WJ, Devesa SS, Kneller RW and Fraumeni Jr JF (1991) Rising incidence of adenocarcinoma of the esophagus and gastric cardia. *JAMA* **65**: 1287–1289

Cameron AJ, Lomboy CT, Pera M and Carpenter HA (1995) Adenocarcinoma of the esophagogastric junction and Barrett's esophagus. *Gastroenterology* **109**: 1541–1546

Cowled PA and Forbes IJ (1985) Phototoxicity in vivo of heamatoporphyrin derivative components. *Cancer Lett* **28**: 111–118

Forssell H (1988) Gastric mucosal defence mechanisms: a brief review. *Scand J Gastroenterol* (suppl.) **155**: 23–28

Jackson MJ and O'Farrell S (1993) Free radicals and muscle damage. *Br Med Bull* **49**: 630–641

Laukka MA and Wang KK (1995) Initial results using low-dose photodynamic therapy in the treatment of Barrett's esophagus. *Gastrointest Endosc* **42**: 59–63

Messmann H, Milkvy P, Buonaccorsi G, Davies CL, MacRobert AJ and Bown SG (1995) Enhancement of photodynamic therapy with 5-aminolaevulinic acid-induced porphyrin photosensitisation in normal rat colon by threshold and light fractionation studies. *Br J Cancer* **72**: 589–594

Murrer LHP, Marijnissen JPA, Baas P, Van Zandwijk N and Star WM (1997) Applicator for light delivery and in situ light dosimetry during endobronchial photodynamic therapy: first measurements in humans. *Lasers Med Sci* **12**: 253–259

Nada O and Kawana T (1988) Immunohistochemical identification of supportive cell types in the enteric nervous system of the rat colon and rectum. *Cell Tissue Res* **251**: 523–529

Panjehpour M, Overholt BF, DeNovo RC, Petersen MG and Sneed RE (1993) Comparative study between pulsed and continuous wave lasers for Photofrin photodynamic therapy. *Lasers Surg Med* **13**: 296–304

Robinson DJ, De Bruijn HS, Van der Veen N, Stringer MR, Brown SB and Star WM (1998) Fluorescence photobleaching of ALA-induced protoporphyrin IX during photodynamic therapy of normal hairless mouse skin: the effect of light dose and irradiance and the resulting biological effect. *Photochem Photobiol* **67**: 140–149

Urano S, Asai Y, Makabe S, Matsuo M, Izumiyama N, Ohtsubo K and Endo T (1997) Oxidative injury of synapse and alteration of antioxidative defense systems in rats, and its prevention by vitamin E. *Eur J Biochem* **245**: 64–70

Van den Boogert J, Van Hillegersberg R, Houtsmuller AB, Siersema PD, De Bruin RWF and Tilanus HW (1997) PDT of Barrett's oesophagus: kinetics and localization of 5-aminolevulinic acid-induced porphyron accumulation in the rat oesophagus. *Proceedings of the International Society for Optical Engineering* **3191**: 214–220

Van Staveren HJ, Beek JF, Ramaekers JWH, Keijzer M and Star WM (1994) Integrating sphere effect in whole bladder wall photodynamic therapy. I. 532 nm versus 630 nm optical irradiation. *Phys Med Biol* **39**: 947–959

Van Staveren HJ, Marijnissen JPA, Aalders MCG and Star WM (1995) Construction, quality assurance and calibration of spherical isotropic fibre optic light diffusers. *Lasers Med Sci* **10**: 137–147

Wang KK (1994) Barrett's esophagus: current and future management. *Compr Ther* **20**: 36–43

Whetsell WO, Sassa S, Bickers D and Kappas A (1978) Studies on porphyrin-heme biosynthesis in organotypic cultures of chicken dorsal root ganglion. I. Observations on neuronal and non-neuronal elements. *J Neuropathol Exp Neurol* **37**: 497–507

Zhou CN, Yang WZ, Ding ZX, Wang YX, Shen H, Fan XJ and Ha XW (1985) The biological effects of photodynamic therapy on normal skin in mice. An electron microscopic study. *Adv Exp Med Biol* **193**: 111–115

 *© Cancer Research Campaign 1999*
